# The Chemistry of Gold Drugs

**DOI:** 10.1155/MBD.1994.107

**Published:** 1994

**Authors:** Peter J. Sadler, Rodney E. Sue

**Affiliations:** 1 Department of Chemistry Birkbeck College University of London Gordon House and Christopher Ingold Laboratories, 29 Gordon Square London WC1H 0PP United Kingdom


### THE CHEMISTRY OF GOLD DRUGS

Peter J. Sadler* and Rodney E. Sue

Department of Chemistry, Birkbeck College, University of London, , Gordon House
and
Christopher Ingold Laboratories, 29 Gordon Square, London WC1H 0PP, UK

### Contents

1. THE CHEMISTRY OF GOLD

1.1. properties of the element

1.2. colloidal gold

1.3. physical methods for the study of gold compounds

1.4. oxidation states

1.5. coordination geometries

2. ANTIARTHRITIC GOLD DRUGS

2.1. gold thiolate and gold phosphine complexes

2.2. ligand exchange reactions

3. GOLD COMPLEXES OF BIOMOLECULES

3.1. gold-albumin complexes

3.2. gold-metallothionein

3.3. gold complexes of other proteins and peptides

3.4. gold complexes with nucleotides

4 POTENTIAL GOLD ANTITUMOUR DRUGS

4.1. Cytotoxic gold phosphines and other complexes

### 1. THE CHEMISTRY OF GOLD

For many centuries gold has occupied a special place in medicine as a potential "cure-all" for diseases. As early as 2500 BC gold was used in Chinese and Arabic medicine. In the 8th century it was advocated as an elixir of youth, and in the Middle Ages gold mixtures were prescribed for a range of conditions.[1] It was not until 1890 that Koch discovered that the gold(I) dicyanide ion had antitubercular activity, although this was subsequently shown to have little benefit for the treatment of the disease.[2] During the search for non-toxic gold(I) complexes with antitubercular activity, gold(I) thiolate complexes were synthesized. These were used extensively during "the golden decade" from 1925-1935 for the treatment of tuberculosis.[1,3] In 1929, Forestier found that gold was effective for the treatment of rheumatoid arthritis,[4] but it was not until 1960 that controlled clinical trials were able to prove the efficacy of gold therapy. Today for the treatment of rheumatoid arthritis, injectable gold(I) thiolates have been supplemented in the clinic by the orally-active gold(I) phosphine complex (auranofin). Auranofin is also being used in the clinic for psoriatic arthritis, juvenile rheumatoid arthritis and is on clinical trial as an antiasthmatic.

There is potential for more extensive use of gold in therapy based on the rational design of new gold compounds. The interest in this area is displayed in the large number of reviews on various aspects, such as those by Shaw (1979),[5] Brown and Smith (1980),[6] Berners-Price and Sadler (1986),[7] Champion et al. (1990),[8] Dash and Schmidbauer (1990),[9] Smith and Reglinski (1991),[10] Parish (1992)[11] and Ni Dhubhghaill and Sadler (1993).[12]

### 1.1. Properties of the element.

Gold, atomic number 79, occurs at the end of the third transition series, with an outer shell electronic configuration of $5d^{10}6s^1$. The enormous stability of Au(0) is a major feature of its chemistry. Gold is a noble metal, easily obtained in a pure metallic form. This has a cubic close-packed structure, each gold atom having 12 nearest neighbors. The yellow metal is malleable and ductile and is resistant to attack by common chemicals and light, and only agents such as cyanide and *aqua regia* (a mixture of nitric and hydrochloric acids) will degrade it. Higher oxidation states of gold are readily reduced to Au(0), and the high strength of Au-Au bonds is another striking feature of its chemistry.[13]

### 1.2. Colloidal gold

Under favourable conditions, solutions of Au(III) can be reduced to colloidal gold. These may be red, blue or violet in colour depending on the method of preparation, mean particle size, and shape. "Purple of Cassius", generated in this manner has been used as a colouring in ceramics for several centuries. Indeed the "potable gold" used in the past as a "cure-all" was obtained by reduction of a solution of gold in *aqua regia* and diluted with essential oils such as oil of rosemary.[1] The surfaces of colloidal gold particles carry a negative charge and so can adsorb strongly to proteins. This has led to the use of colloidal gold as a cytochemical marker in electron microscopy for protein binding studies.[14] For example, colloidal gold labelled with antibodies can be used to probe antigenic sites on cell surfaces. Imaging of the liver can be carried out using radiolabelled $^{198}Au$ administered by injection. Macrophages also take up gold particles by phagocytosis. Recently, ultrasound has been used to give small (<10 nm) particles,[15] and reduction with $[P(CH_2OH)_4]Cl$ gives even smaller particles (1.5 nm).[16]

### 1.3. Physical methods for the study of gold compounds

There are few methods for the direct probing gold in gold complexes. These include $^{197}Au$ Mössbauer[17] and X-ray absorption spectroscopy.[18] $^{197}Au$ (100% abundance) NMR has not been useful for examining gold complexes since the nucleus is quadrupolar (I=3/2) with a large quadrupole moment and low gyromagnetic ratio. Only in the most symmetrical compounds would the $^{197}Au$ NMR signal be expected to be sharp enough to observe, and then only at high concentrations. No useful chemical studies have been done, and nuclear quadrupole resonance has also not found wide applications for gold. Single crystal X-ray diffraction give useful structural data if suitable crystals can be obtained, indeed gold derivatives are often used to solve the phase problem in protein crystallography. Extended X-ray absorption fine structure (EXAFS; e.g. $L_{III}$ edge) and wide angle X-ray scattering (WAXS) have been used to study the gold coordination sphere in a number of complexes. Electron paramagnetic resonance (EPR) has been used to study several Au(II) compounds[13] but the biologically important oxidation states of Au(I) and Au(III) are not EPR active. Table 1 summarizes the information gained from these techniques. Gold complexes are mostly studied by indirect means such as NMR, vibrational and electronic spectroscopy.

### Table 1

Physical Methods for The Study Of Gold Compounds

| Technique | Information | Comments |
|---|---|---|
| X-ray crystallography | bond lengths and angles | need crystals<br>3-dimensional structure |
| X-ray spectroscopy - EXAFS, WAXS | bond lengths, distances to nearest neighbours, number and type of Au-bonded atoms, oxidation state | requires synchrotron radiation, can be used for solutions and amorphous solids, difficult to distinguish N from O, and P from S or Cl |
| Mössbauer spectroscopy | oxidation state, total electron density at Au (isomer shift) and symmetry (quadrupole splitting) | short source half-life ($^{197}$Pt, 18 h), low temp. required (4K), for solids only |
| Nuclear magnetic resonance spectroscopy (NMR) | number and type of coordinated ligands, conformation, ligand exchange rates (dynamics) | probes ligand rather than Au, usually $^{1}$H, $^{13}$C, $^{31}$P, $^{15}$N, generally need solutions, although solid state spectra are possible, can be used to probe metabolism in biological media or intact organisms |
| Electronic absorption spectroscopy, and circular dichroism (CD) | electronic energy levels and symmetry (CD) | absorptions are often weak in the visible region, charge -transfer absorptions in the UV region |
| Infrared and Raman spectroscopy | ligand, and Au - ligand vibrational frequencies | ligand bands often do not change substantially on coordination, bands due to Au - ligand vibrations can be difficult to assign (Au - Cl, S *ca.* 340 $cm^{-1}$, Au - Br *ca.* 230 $cm^{-1}$, Au - I *ca.* 180 $cm^{-1}$) |
| Mass spectrometry | molecular mass of parent and fragments | compound must be introduced into the ion source, fragmentation pattern can be obtained |

Total gold determinations are usually carried out by atomic absorption spectroscopy (AAS) where the detection limit is *ca.* 0.5 ppm, or, more recently, by inductively coupled plasma-mass spectrometry (ICP-MS). The use AAS to monitor serum gold levels has greatly improved the safety of use of gold drugs since the 1970's. Recently the metabolism of auranofin has been studied by HPLC coupled to ICP-MS, the detection limit of which is four orders of magnitude lower than AAS. This is approximately the concentration of gold in the blood and urine of patients treated with the drug.[19]

1.4. Oxidation states

Au(I), outer electronic configuration $5d^{10}$, is by far the most important form of gold biologically, and most gold drugs contain gold in this oxidation state. It is possible that Au(I) can be oxidized to Au(III) *in vivo*, e.g via the myeloperoxidase system of white cells.[20] Au(III) may be responsible for some of the toxic side-effects of Au(I) drugs (Section 3.4). Au(II) could be an important intermediate in biological reactions, but has not yet been recognized as such, while Au(V) is not likely to be accessible *in vivo*.

Whife Au(I) dominates the biochemistry of gold compounds, it is important to note that the gold must be stabilized by π-acceptor ligands (those capable of accepting back-bonding from the metal) because there is a tendency to disproportionation to Au(III) and colloidal Au(0), particularly in aqueous solution. The oxidation state diagram illustrates the high thermodynamic stability of Au(0). The Au(III) complexes generally have high potentials indicative of their oxidizing properties. Many of the Au(I) complexes have $E^o$ values that lie above a line drawn from the potentials of the corresponding Au(III) complexes and Au(0) indicating that the complexes are unstable with respect to disproportionation to Au(III) and Au(0). The low points for $[Au(CN)_2]^-$ and $[Au(S_2O_3)_2]^{3-}$ indicate stabilization of Au(I) for these complexes. The $E^o$ for $[Au(H_2O)_2]^+$ is a calculated value[21] (Figure 1).[139]

An approximate order of thermodynamic stability[22] for some Au(I) complexes with biologically-important ligands is:-

$$CN^- \sim CysS^- \sim PR_3 >> Met\text{-}S\text{-}CH_3 \sim His(=N\text{-}) > Cl^- >> COO^-$$

and for halides:-

$$I^- > Br^- > Cl^- >> F^-$$

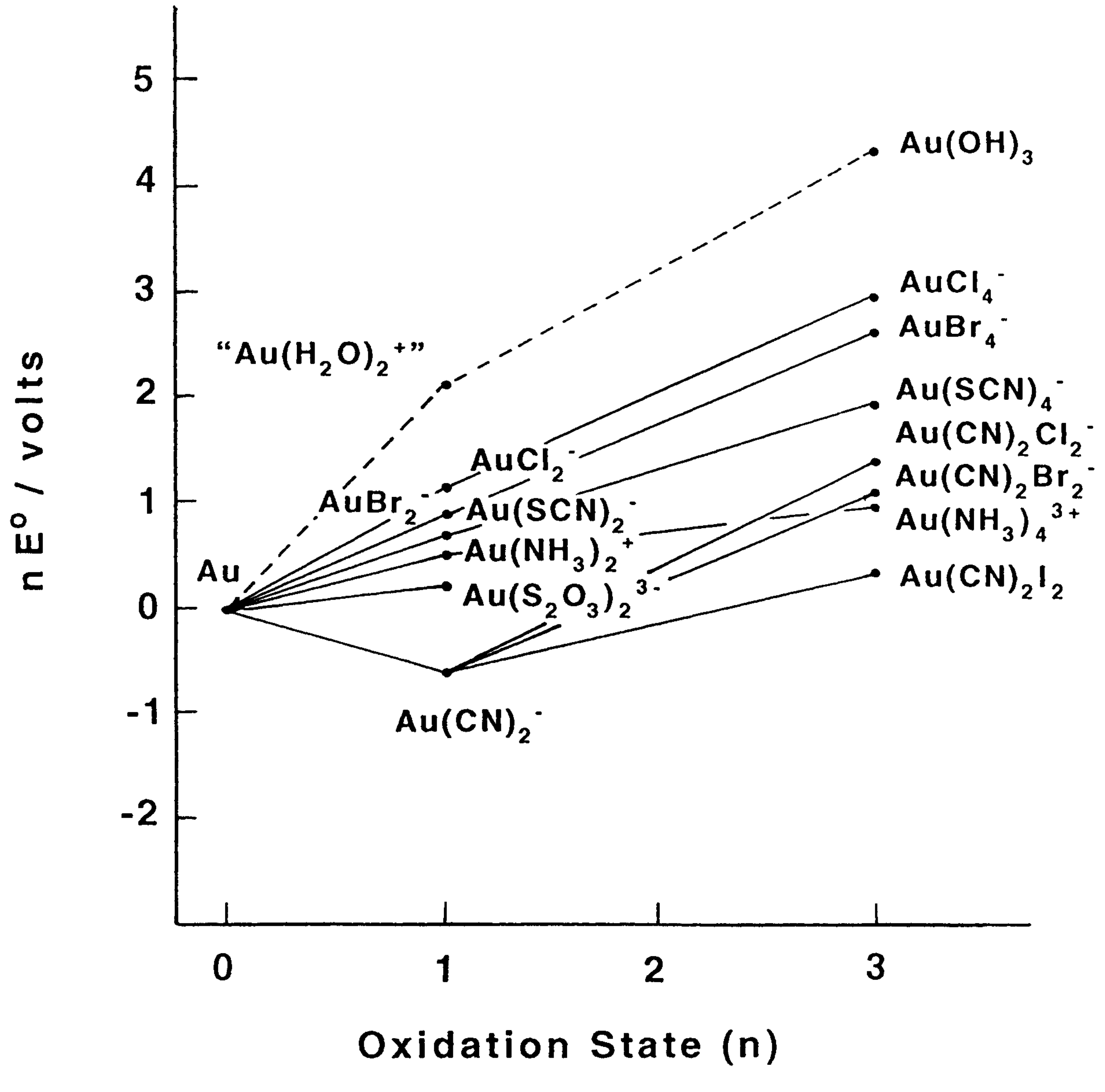

**Figure 1**: Oxidation state diagram for gold (adapted).[139]

Generally the Au(I) complexes are prepared from Au(III) precursors such as $AuCl_4^-$, and there are a variety of methods available, such as reduction with excess phosphine or thiodiglycol. Electrolytic methods for producing Au(I) often have the advantage of giving fewer by-products and allow a ready choice of counter-anion.[23]

1.5. Coordination geometries

X-ray crystallography has shown that Au(I) can adopt coordination numbers of two, three, and four. Linear two-coordination is the most common, often with weak additional bonds (especially Au···Au contacts) in the solid state. A recent analysis of Au···Au interactions in the solid-state shows a large number of contacts in the range 2.50 - 4.00 Å. For comparison, the Au···Au distance in metallic gold is 2.89 Å and the van der Waals distance is 3.60 Å, illustrating the tendency for both bonding and non-bonding Au-Au intermolecular contacts.[24] For Au(III), square-planar four coordination is the most common, but five- and six-coordination are known. Examples are shown in Figure 2.

2. ANTIARTHRITIC GOLD DRUGS

2.1. Gold thiolate and gold phosphine complexes

Since Landé first suggested the use of aurothioglucose (Solganol),[25] a number of Au(I) thiolato complexes have been used as injectable drugs in antiarthritic therapy, these have included sodium aurothiosulfate (Sanochrysin), sodium aurothiomalate (Myocrisin), sodium aurothiopropanol sulfonate (Allocrysin) and the Au(I) complex of 4-amino-2-mercaptobenzoic acid (Krysolgan), Figure 3. The thiolate complexes are polymeric in the solid state and in solution, forming rings or chains.

Of these complexes, only Sanochrysin has had its structure determined by X-ray crystallography. The $[Au(S_2O_3)_2]^-$ ion is linear and two-coordinate with a bond angle of 176° and Au-S distance of 2.28 Å while the nearest Au neighbours are 3.30 Å away.[26]

Aurothiomalate was developed with many other potential drugs in the 1920's during the search for less toxic antitubercular agents to replace $[AuCN_2]^-$.[22] The structures of both aurothiomalate and aurothioglucose have been studied by EXAFS since neither has been crystallized.[27,28]

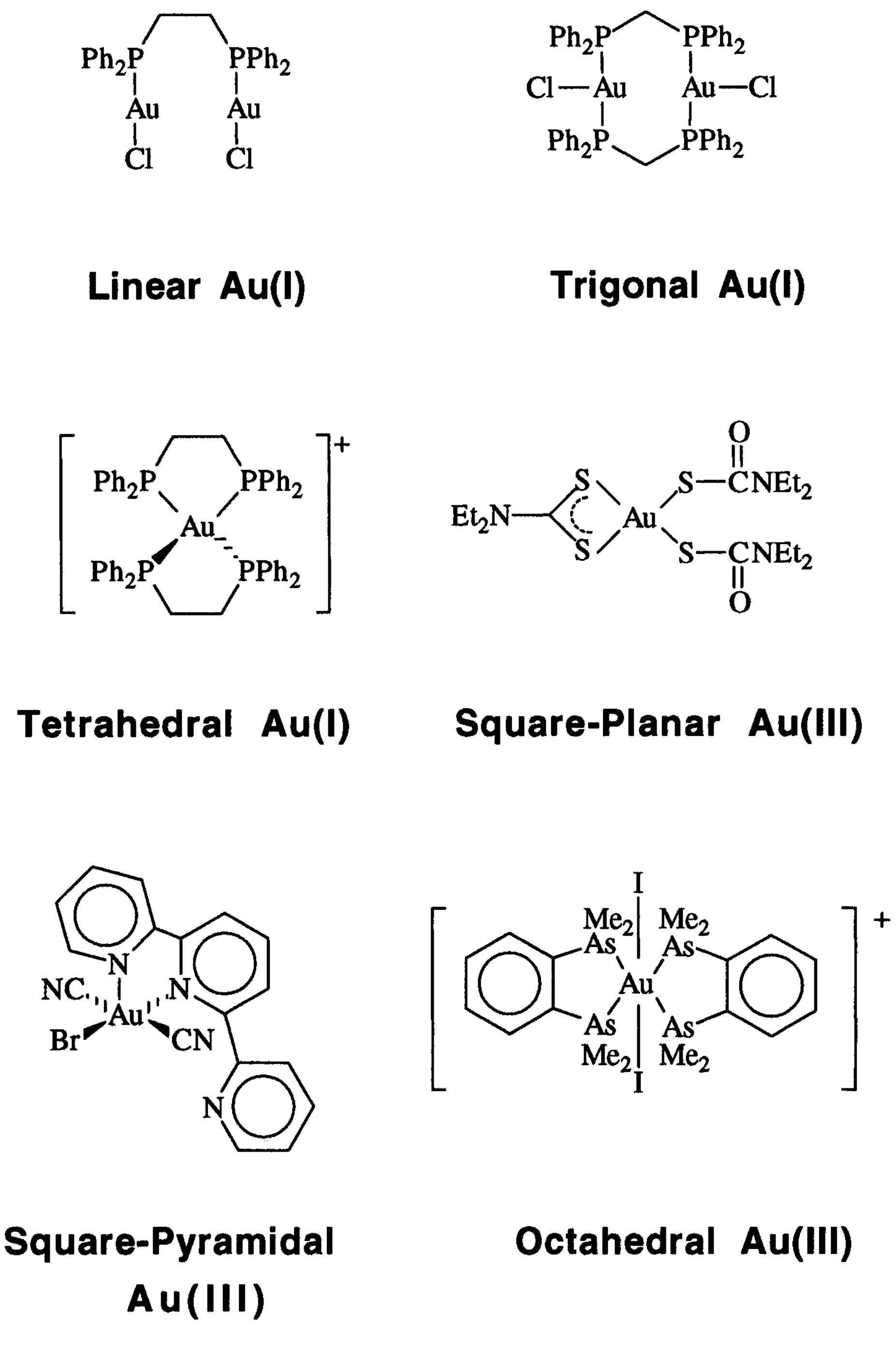

**Figure 2**: Typical structures of Au(I) and Au(III) complexes.

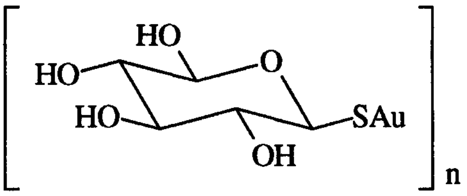

**aurothioglucose**
**(Solganol)**

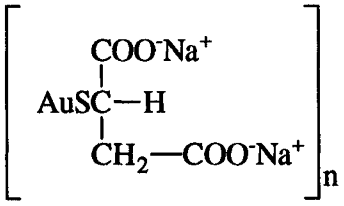

**sodium aurothiomalate**
**(Myocrisin)**

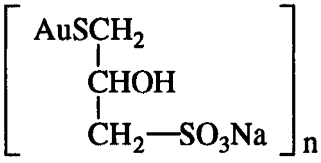

**sodium aurothiopropanol sulfonate**

**(Allocrysin)**

$$[O_3S{-}S{-}Au{-}S{-}SO_3]Na_3$$

**sodium aurothiosulfate**
**(Sanocrysin)**

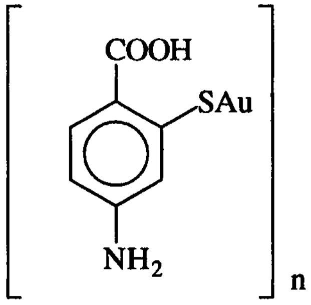

**gold(I) complex of 4-amino-2-mercaptobenzoic acid**
**(Krysolgan)**

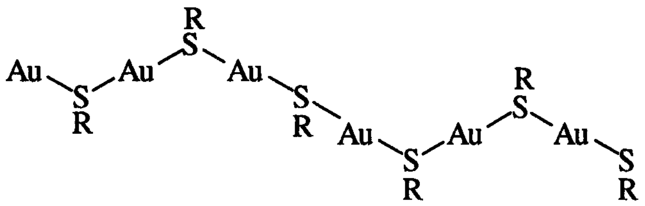

**polymeric chains**

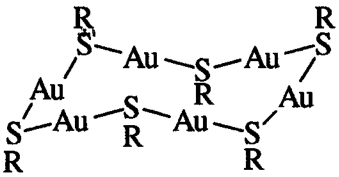

**rings**

**Figure 3**: Injectable gold antiarthritic drugs and gold(I) thiolate chains and rings.

In both cases the gold is bound to two sulfur atoms at a distance of 2.37 Å giving polymers with bridging thiolate sulfurs. Cyclic hexamers or pentamers are possible,[29] but WAXS measurements, which allow detection of Au···Au contacts (of 3.35, 5.8 and 8.1 Å), suggest that a linear hexamer structure is the more likely as shown in Figure 3.[30] Such structures require that the thiolate be present in stoichiometric excess over gold in these complexes, which is usually the case (*ca.* 9% molar excess).[31]

In solution the structure of aurothiomalate is highly dependent on the charge on the carboxylate groups. At low ionic strength the conformation of the ligand is similar to that of free thiomalate, while at higher ionic strength the structure is more compact and a number of conformations can be detected by NMR.[29] EXAFS studies of solutions of aurothiomalate and aurothioglucose show that Au(I) is coordinated to 2 sulfur atoms at distances of 2.29 Å and 2.30 Å, respectively.[28] The kinetics of the folding and unfolding of the various polymeric forms of aurothiomalate have been analyzed using electronic absorption spectroscopy.[32] It is notable that the uptake of gold is greater in inflamed tissue than for non-affected tissue.[33]

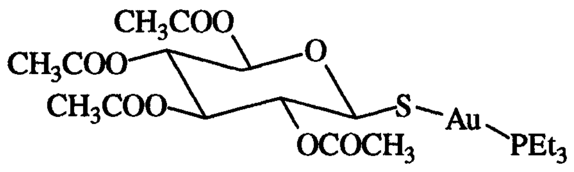

**Figure 4**: The orally-active antiarthritic drug, auranofin.

One of the problems with traditional chrysotherapy are the side-effects, the most severe being exfoliative dermatitis, bone marrow suppression and nephrosis. Auranofin resulted from a search for an orally-active drug to overcome these problems and those associated with painful intramuscular injection of gold thiolates.[2] Several gold(I) phosphine complexes were found to have oral antiinflammatory activity in animal models, including $Et_3PAuCl$ and $(Et_3P)_2AuCl$, but diarrhoea was a troublesome side effect.[34,35] Eventually the tetraacetylthioglucose derivative, auranofin (Figure 4) was approved by the FDA in 1985. X-ray crystallography shows that auranofin is monomeric with linear 2-coordinate Au(I) with Au - P and Au -S bond lengths of 2.259 Å and 2.293 Å, respectively, and with a P - Au - S angle of 173.6°[36] and $^1H$, $^{13}C$ and $^{31}P$ nmr show that the structure is similar in solution.[37,38] The gold phosphine complex has similar geometry in the solid state with the Au - P and

Au - Cl bond lengths of 2.232 Å and 2.305 Å, respectively, and a P - Au - Cl angle of 178.5°.[39]

Auranofin is a lipophilic complex and blood serum levels are maintained with a daily dose as opposed to the weekly or monthly injections of aurothiomalate. Whereas aurothiomalate is almost immediately taken into the blood stream and distributed to all parts of the body, auranofin is absorbed only slowly. There is little difference in gold distribution 24 h after administration of the two complexes. It should be noted that if solid auranofin is administered, only 20-25% is absorbed and so the effective dose is much lower. However, if solutions of auranofin are administered (e.g. in ethanol) then absorption is almost complete.[40]

Auranofin has been shown to be an inhibitor of the incorporation of $^{3}H$-thymidine and $^{14}C$–amino acids in mitogen-stimulated human lymphocytes, i.e. the precursors to DNA and protein synthesis.[41] Inhibition of protein kinase C, which is important in the transmission of extracellular signals, has also been reported, probably by interaction with thiol groups.[42,43] Au(I) thiolate drugs may also be involved with suppressing the production of reactive oxygen species. Phagocytosis may result in the generation of superoxide ion and it has been shown that this can be oxidized to singlet oxygen. This in turn is capable of peroxidation of unsaturated fatty acids, and may be involved in the inflammatory process. Auranofin also deactivates singlet oxygen with a quenching constant of the order of $10^7$ $M^{-1}s^{-1}$.[44] Both auranofin and myocrisin have been implicated in the control of oxidative damage in rheumatoid arthritis.[45]

Gold phosphine complexes of thiobenzoic acid ($Et_3PAuSCOPh$) and substituted thiophenols (*o*-$HOOCC_6H_4SAuPEt_3$ or *o*-$H_2NC_6H_4SAuPEt_3$) have also shown antiarthritic activity in rats, but have not been used clinically.[46] Gold yeast (containing 0.5 % gold by weight), obtained by growing yeast on Au(III), is also found to be effective against adjuvant arthritis.[47]

### 2.2. Ligand Exchange

Thiolate exchange reactions of aurothiomalate are facile via associative mechanisms and three-coordinate intermediates, and the polymers are degraded eventually into monomeric bisthiolato complexes $[Au(SR)_2]^-$. The thiols with the lowest $pK_a$'s form the most stable complexes; an approximate order of stability for complexes with some biologically important ligands is:-[48]

cysteine methyl ester, D-penicillamine > β-D-thioglucose > N-acetylcysteine
> glutathione, thiomalate, mercaptoacetate.

Thiols such as D-penicillamine (β,β-dimethyl-D-cysteine) and 2,3-dimercaptopropanol are sometimes prescribed to remove gold from the body in cases of toxicity because of the high stability of their complexes with gold.[49,50]

Such thiolate exchange reactions play a role in the transport and metabolism of gold drugs. Aurothiomalate is largely transported around the body on serum proteins such as albumin. Mössbauer and EXAFS studies show that the thiomalate is exchanged for cysteine-34 in albumin:-[51]

$$\text{Alb-Cys-34-SH} \quad + \quad 1/n[\text{Autm}]_n \longrightarrow \text{Alb-Cys-34-S-Autm}$$

It is notable that the $pK_a$ of Cys-34 in albumin is low (< 5).

Smith et al. have used resonance Raman, in conjunction with 5,5-dithiobis(2-nitrobenzoic acid), Ellman's reagent, and nmr spectroscopy to probe membrane thiols of red blood cells, and have shown that these thiols are targets for aurothiomalate.[51] $^{15}N$ Nmr has also been used to study the interaction of $C^{15}N^-$ with both aurothiomalate and aurothioglucose, and in both cases the cyanide ion immediately binds to gold, forming $[RSAuCN]^-$ complexes. These rearrange to $[Au(CN)_2]^-$ and $[Au(SR)_2]^-$ species. This may influence the uptake of these drugs into the red blood cells by depolymerising the gold drugs and allowing them to cross the red cell membrane [52]

Auranofin is readily deacetylated under acidic conditions such as those in the stomach and triethylphosphineAu(I)thioglucose has been detected after passage of the drug through the intestinal wall.[54] $^{31}P$ nmr spectroscopy is useful for probing interactions between gold phosphine complexes and biofluids, cells and proteins.[38,55] In $R_3P$-Au-SR complexes, the $^{31}P$ chemical shift is dependent on the $pK_a$ of the thiolate (SR) in the *trans* position.[56]

Auranofin and related complexes are readily taken up into red cells where the binding sites are glutathione and haemoglobin (Cys-β-93). $Et_3P$-Au-Cl binds preferentially to the free Cys thiolates of haemoglobin and albumin, but when these are saturated, then weaker binding to His residues occurs.[38,55,57,58] Histidine (His) binding is probably responsible for the rather unusual low-spin state to high-spin state change which this complex can induce in cytochrome c.[59]

Studies of the metabolism of triply-radiolabelled auranofin ($^{32}P$, $^{195}Au$, $^{35}S$) have shown that excretion of the sulfur and phosphorus occur faster than excretion of gold,[60] consistent with the displacement of the (acetylated)thioglucose ligand by natural thiolate ligands *in vivo*, and subsequent displacement of triethylphosphine and its rapid oxidation to $OPEt_3$. The latter process may be accompanied by reduction of a protein disulfide bond.[38,61]

Exchange reactions involving cyanide are also of physiological importance. Smoking has been shown to increase the uptake of gold into red blood cells. This is attributable to inhalation of HCN in smoke.[62] Cyanide reacts with aurothiomalate to form the mixed ligand complex $[Au(CN)(tm)]^-$, and then $[Au(CN)_2]^-$.[63] The latter complex is readily taken up into red cells whereas aurothiomalate is not. $[Au(CN)_2]^-$ has been detected as a metabolite in the urine of patients treated with both aurothiomalate and auranofin;[19] for example 36% of the gold in a urine sample of a patient (smoker) treated with Solganol was present as $[Au(CN)_2]^-$. $[Au(CN)_2]^-$ may be a common metabolite for all gold antiarthritic complexes. Since cyanide is also a natural metabolite (converted into $SCN^-$ by rhodanese in the liver) it may play a more important role in the action of gold (and some other metallodrugs) than has previously been realized. This requires further investigation.

### 3. GOLD COMPLEXES OF BIOMOLECULES

### 3.1. Gold-albumin complexes

Over 80% of the gold in circulation in the body after administration of gold drugs is bound to the protein albumin (M 66.5 kDa).[64] The protein contains 35 cysteine residues, all but one of which are oxidised as disulfide bridges. The remaining cysteine, Cys-34, is present in the reduced form (SH) in 60-70% of albumin molecules while 30 - 40% is a mixed disulfide with free cysteine or glutathione. Albumin-bound gold is probably then transferred into cells. The high affinity of Cys-34 for Au(I) is consistent with its reported low $pK_{SH}$ (ca.5). EXAFS and Mössbauer spectra of albumin after modification with aurothiomalate suggest the presence of Alb-Cys-34-S-Au-tm with Au-S distances of 2.28 Å.[51] Au-P and Au-S distances of 2.29 Å have been determined by EXAFS for Alb-Cys-34-S-Au-

$PEt_3$ obtained by treatment of albumin with auranofin. Triple radiolabelling experiments ($^{3}H$, $^{195}Au$, $^{14}C$) confirm that auranofin binds to albumin with release of the tetraacetylthioglucose ligand.[66] In accordance with the $pK_{SH}$ values, Au(I)$PEt_3$ readily transfers from haemoglobin Cysβ93 to albumin Cys-34.[57] $^{1}H$ Nmr studies suggest that the binding of $Et_3PAu^+$ to Cys-34 of albumin influences the environment of His3 at the N-terminus. Since this is the site of Cu(II) transport, and copper has been implicated in the aetiology of rheumatoid arthritis, this finding could have a wider significance. *In vitro*, reactions between $Et_3PAuSR$ and albumin lead to the release of SR, which in turn can reduce the Cys-blocked albumin in the solution (ca. 40% for bovine serum albumin) with concomitant production of disulfides Cys-SR, and Cys-Cys (e.g SR = thioglucose, tetraacetylthioglucose).[67] $^{31}P$ Nmr studies show that treatment of albumin with $Et_3PAuCl$ gives a Au(I) complex with phosphorus and sulfur ligands ($\delta^{31}P$ 42.0 ppm), further evidence for complexation at Cys-34. Additional peaks at $\delta^{31}P$ 34.4 and 30.3 ppm are indicative of Au(I) binding to other sites such as histidine and methionine;[55] there may also be a conformational change on gold binding.[58] In addition, there is evidence for the sulfur-bridged species Alb-Cys-S-$(AuPEt_3)_2^+$ ($\delta^{31}P$ 35.6 ppm).[68] Once $Et_3Au^+$ is bound to Cys-34 of albumin, the phosphine can be displaced by other ligands such as $CN^-$ with release of the phosphine, this may influence the rate of gold metabolism in smokers.[69] GC-MS studies with $^{17}O$ nmr studies suggest that albumin disulfides are reduced by oxidation of the free phosphine.[70] Indeed it has been shown that treatment of albumin with $(Et_3P)_2AuCl$ results in the formation of Alb-Cys-S-$AuPEt_3$ and free $Et_3P$ which is in turn oxidised by disulfide bridges in albumin. The free thiols formed may then bind more gold drug. This results in a family of gold-albumin complexes with variation in the position of gold binding determined by which of the disulfide bridges is reduced.[71] Tetraacetylthioglucose also stimulates $OPEt_3$ production by exchange reactions:-[72]

$$\mathrm{Tg(Ac)_4} + \mathrm{Alb\text{-}Cys\text{-}S\text{-}Au\text{-}PEt_3} \rightleftharpoons \mathrm{Alb\text{-}Cys\text{-}S\text{-}Au\text{-}S\text{-}Tg(Ac)_4} + \mathrm{Et_3P} + \mathrm{H^+}$$

$$\mathrm{Et_3P} \longrightarrow \mathrm{Et_3P{=}O}$$

This is an illustration of the *trans* effect, resulting in labilization of the phosphine and although the first step is reversible, the second step is irreversible and results in gradual loss of the phosphine. Interestingly the effect is observed only for tetraacetylthioglucose and not for thioglucose or glutathione.

### 3.2. Gold Metallothionein.

Metallothionein (Mt) is a protein of 61 residues of which 20 are cysteine residues. It is thought to have a role in intracellular storage of Cu(I) and Zn(II) and may also play a part in the transport and detoxification of other metal ions (e.g. Hg(II) and Cd(II)). When aurothiomalate reacts with metallothionein, Au(I) displaces Zn(II) and Cd(II).[73] If the protein is in excess then thiomalate is completely displaced, but if the drug is in excess, thiomalate (tm) may be retained as Mt-Cys-Au-tm. Up to 20 Au-tm bonds can form, consistent with gold binding to each of the cysteine residues with Au(I) - S distances of 2.30 Å as shown by EXAFS. In contrast, auranofin does not displace zinc or cadmium, although it will bind to the apoprotein. $Et_3PAuCl$, on the other hand, binds strongly to metallothionein indicating the ease of displacement of chloride compared to thiolate sulfur.[66,74] Cultured human epithelial cells can be stimulated to metallothionein synthesis by exposure to $[AuCl_4]^-$.[75] Auranofin also induces metallothionein synthesis in Chinese hamster ovary cells via activation of gene transcription, and appears to provide a gold resistance mechanism.[76] *In vivo*, administration of Au(I) to rats leads to Mt-bound gold. The levels increase rapidly for 12 hours and then more slowly for the next 5 days, before declining.[77]

### 3.3. Gold complexes of other proteins and peptides

Under highly inflammatory conditions, as in arthritis, there are likely to be high levels of strong oxidants available, e.g. $H_2O_2$, $ClO^-$, $IO^-$ and some of the side-effects associated with Au(I) antiarthritic drugs may be due to *in vivo* oxidation to Au(III). Gold(III) peptides could be responsible for lymphocyte activation processes. There are few well-established structures of Au(III)-peptide complexes. Au(I) binds predominantly to cysteine sulfur in peptides, and much more weakly to methionine sulfur and histidine nitrogen. Au(III) on the other hand binds strongly to N and O side-chains of peptides and deprotonated amide nitrogens. The latter type of binding is shared by the other square-planar ions Cu(II), Ni(II), Pd(II), and Pt(II). Au(III) tends to oxidize methionine and cysteine sulfurs.[78]

In $[Au(Gly\text{-}L\text{-}His)Cl]^+$ the peptide behaves as a tridentate ligand with square-planar Au(III) coordinated to the glycyl amino group, the deprotonated peptide N and the N3 of the His imidazole ring. The Au-N(peptide) bond is slightly shorter than the other two Au-N bonds (1.94 Å cf. 2.00 Å). This monomer was crystallized at pH 1.5-2, and at pH 6 to 7 a tetramer $[Au(Gly\text{-}L\text{-}His)]_4$ is formed. Here the fourth coordination site is occupied by a bridging N from the deprotonated imidazole ring instead of chloride. The four Au atoms form a distorted tetrahedron with a $C_2$ axis in the centre of a saddle arrangement (Figure 5).[79]

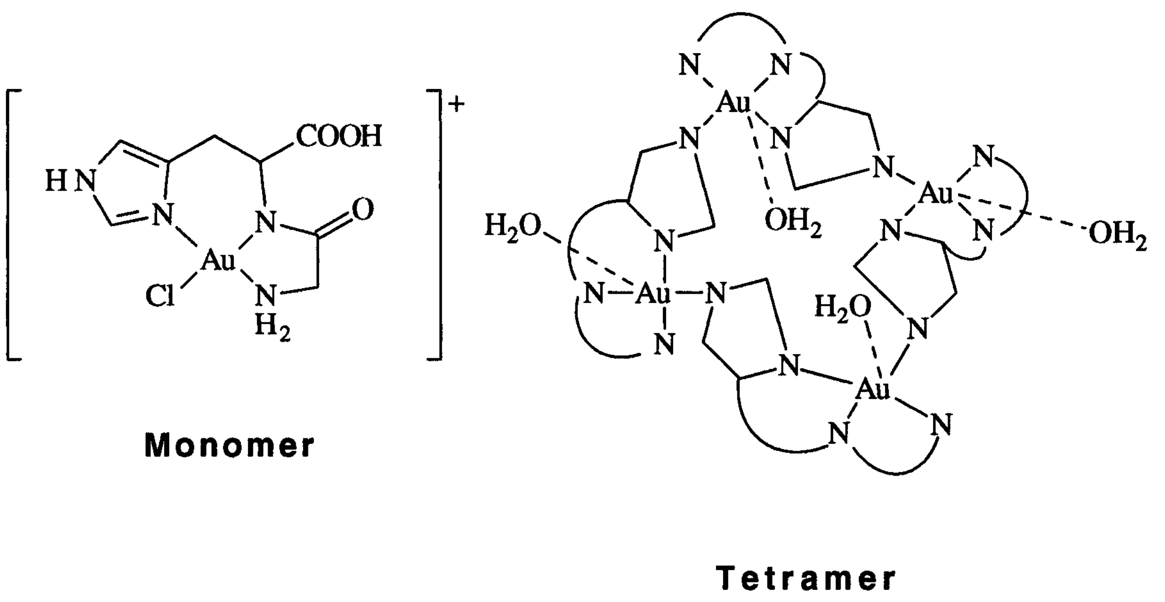

**Figure 5**: Structures of $[Au(gly\text{-}L\text{-}his)Cl]^+$ And $[Au(gly\text{-}L\text{-}his)]_4$ as determined by Wienken et al., 1992.[79]

There are several reports of gold amino acid derivatives, (L-cysteinato)gold(I) and (D-penicillaminato)gold(I) have been prepared and studied electrochemically,[80] and a number of salts of the form $[H_3NCHRCO_2R']^+[AuCl_4]^-$ have been prepared. Of these, only the methyl alaninium salt (Figure. 6) has been studied crystallographically, the structure is unexceptional with little interaction between the amino acid and the $[AuCl_4]^-$ ion.[81] Triphenylphosphine-(N-benzoyl-L-alaninato)gold(I) has also been crystallized (Figure. 6). As expected, the gold is linearly coordinated by the phosphine (Au - P, 2.22 Å) and the amino acid carboxlate group (Au - O, 2.07 Å).[82] This is one of the few examples of Au - O bonds.

Gold-protein complexes are often prepared by X-ray crystallographers (by soaking crystals in solutions of gold complexes) to solve the phase problem. For example, the antibiotic valinomycin (a cyclododecadepsipeptide) was shown to be held in the proper conformation for potassium binding with hydrogen bonds, by using the tetrachloroaurate salt to solve the crystal structure.[83] Ions such as $[Au(CN)_2]^-$ often bind electrostatically, for example to the $NAD^+$ binding site on liver alcohol dehydrogenase.[84] However, details of gold binding are often not reported.

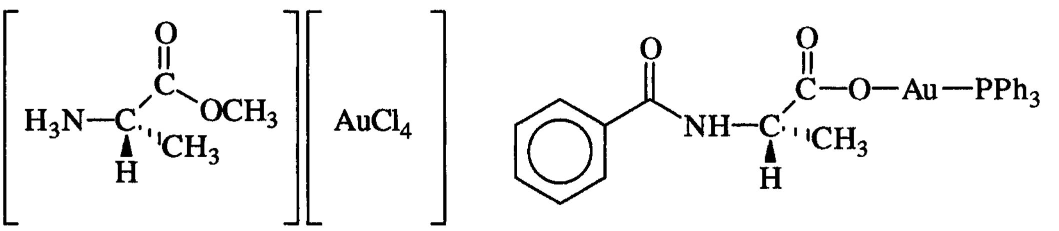

**Methyl-L-alaninium Tetrachloroaurate** **Triphenylphosphine-(N-benzoyl L-alaninato)gold(I)**

**Figure 6**: Gold complexes of amino acids which have been determined by X-ray crystallography.

Schuhmann et al.have reported that Au(III) induces antinucleolar autoantibodies in animals. They propose that Au(I) drugs produce toxic side-effects via oxidation to Au(III), for example in phagolysozomes (aurosomes). Self-proteins or peptides become modified by Au(III) and give rise to sensitized T-cells and adverse immunological reactions.[85] However, Romagnoli et al. have found that lymphocytes of patients who develop toxic skin reactions to gold, proliferate *in vitro* when challenged with different gold compounds. Unlike the findings of Schuhmann et al., they found that gold specific T-cells recognized Au(I) and not Au(III).[86]

Zinc fingers are DNA-binding proteins, typically with two histidines and two cysteines as Zn(II) ligands. These may be targets for gold binding.[87] Indeed aurothiomalate has recently been found to inhibit binding of the progesterone receptor (PR) to its DNA response element.[88] Displacement of Zn(II) by Au(I) is likely to disrupt DNA binding since Au(I) is likely to adopt a linear coordination in the flexible loops of this protein rather than the tetrahedral stereochemistry adopted by Zn(II).

There are a number of reports of gold compounds inhibiting DNA polymerase,[89] RNA and protein synthesis,[90] adenosine triphosphatase[91] and a number of enzymes.[92] The toxicity of Au(III) compounds may also involve oxidation of protein disulfides by Au(III).[78] On the other hand, synthesis of a 32 kD human stress protein has been reported to be induced by auranofin.[93]

3.4. Gold complexes with nucleotides

Nucleotides with only nitrogen or oxygen donors would be expected to bind only weakly to Au(I). This is consistent with the lack of mutagenicity and carcinogenicity of most gold complexes, and is an obvious advantage for gold drugs. Instead, the cytotoxicity of gold(I) phosphine complexes has been attributed to either the phosphine moiety or to interactions of the gold phosphine complexes with proteins such as DNA polymerase. Indeed it has been shown that auranofin does not interact with pBR322 DNA, although the analogous $Et_3PAuX$ complexes (X = Cl, Br) do bind to DNA, under certain conditions (pH 9.5), with a preference for guanine and cytosine. This can be inhibited by the addition of a thiosugar, and illustrates the preference of Au(I) for softer donors such as sulfur.[94, 95] However, Au(III) does bind strongly to nitrogen and oxygen donors and a number of Au(III) interactions with nucleotides have been reported. For example, $Et_3PAuBr_3$ binds to *Hind III/NciI*, a 139 base-pair restriction fragment from pBR322 and this inhibits cleavage at guanine N(7).[96] In addition, there have been a number of reports of interactions between nucleobases and nucleosides with both Au(I) and Au(III). These include complexes of the form $[Au(III)(CH_3)_2(nucl)Cl]$, where nucl = adenosine, cytidine or guanosine. In all of the complexes the Au(III) has square-planar geometry with the gold bound to N(7) of guanosine, and N(3) of cytidine, while for adenosine the gold may be bound to N(7) or the amino group.[97] Thermolysis of these complexes results in elimination of the nucleoside.[98] Au(I) complexes include the gold phosphine complexes $[Au(I)(PR_3)(nucl)]NO_3$, where R = phenyl, *o*-anisyl, *p*-anisyl; nucl = guanosine, adenosine or cytidine, where the nucleoside binding sites are again thought to be N(7), N(7) and N(3), respectively.[99]

Uridine and uracil complexes have been more elusive; the primary reaction between uridine or uracil and $[AuBr_4]^-$ gives the corresponding 5-bromopyrimidine and/or the 5-bromo-6-hydroxypyrimidine.[100, 101, 102] However, the related Au(III) complex of 6-amino-1,3-dimethyl-5-phenylazouracil (DZH), $[Au(DZ)Cl_2]$, has been prepared and is thought to have square-planar geometry with the ligand *cis*-coordinated through the nitrogen of the azo group bonded to the phenyl ring and the deprotonated amino group, with the remaining sites occupied by 2 chlorine atoms.[103] The 5-diazouracil (5-du) complex of Au(III), $[Au(5\text{-}du)_2Cl_2]Cl{\cdot}HCl$, has also been prepared and shows antitumour activity.[104]

Crystallographic studies include inosinium tetrabromaurate dihydrate,[105] 9-ethylguaninium tetrachloroaurate hydrate,[106] tetrakis(1-methyluracilium)sodium tetrachloroaurate and $[Na(1\text{-}MeTh)(H_2O)_4][AuCl_4].1\text{-}MeTh.2H_2O$ (1-MeTh = 1-methylthymine).[107] In each case Au(III) is square-planar with little association between the tetrahaloaurate ion and the base. In contrast, gold is bound to the nucleobase in trichloro(1-methylcytosinato)gold(III). This complex is square-planar with the Au(III) bound to N(3) of 1-methylcytosine with an Au-N(3) distance of 2.031Å; the angle between the planes of 1-methylcytosine and the $AuCl_3$ is 85°, which should minimize steric replusion.[108] $^1H$ Nmr studies suggest that there is similar binding in trichloro(cytosinato)gold(III).[109] The gold geometry is also square-planar in $[Au(2,2'\text{-bipyridine})(1\text{-methyluracil})_2]ClO_4.4H_2O$, here there are 4 nitrogen donors with the nucleobases arranged in a head-to-tail fashion.[110] Complexes of 3′-azido-3′-deoxythymidine (HAZT), $[R_3PAu(AZT)]$ exhibit antiinflammatory activity for R = $CH_3$, and anti-HIV activity for R = $CH_3$, Ph. The trimethylphosphine complex has been crystallized and contains linear 2-coordinate Au(I).[111] Similar geometry is observed in the phosphine gold thiouracil complexes, $[R_3PAu(2\text{-thiouracil})]$, R = ethyl, phenyl.[112, 113] Structures of these complexes are shown in Figure 7.

Chloro(pyridine)gold(I) and trichloro(pyridine)gold(III) both appear to react with a number of different conformations of pBR322 DNA to produce inter-strand cross-links and single-strand breaks as does $Et_3PAuCl_3$. On the other hand the Au(I) complex, $Et_3PAuCl$ binds without the formation of cross-links. The amount of cross-linking can be reduced by the addition of thiols such as 2-mercaptoethanol, again illustrating the strength of Au(I)-P and Au(I)-S bonds.[94, 114]

Gold(III) nucleotides have been prepared from $AuCl_4^-$ and adenine nucleotides. If the only cations present are $Na^+$ or $K^+$ they are soluble but can be precipitated with ethanol. Little is known about their structure, although they may be polymeric with coordination to $NH_2$(N6) and N7 of the adenosine.[115] Probable binding sites for Au(III) with nucleotides have been suggested to be N(1)/N(7) for adenine, N(7) or C(6)O of guanine, N(3) of cytosine and N(3) of thymidine which are analogous to the binding sites for the isoelectronic Pt(II) ion.[95, 116, 117] Further work in this area is required to fully understand the impact of gold binding to nucleotides on drug action.

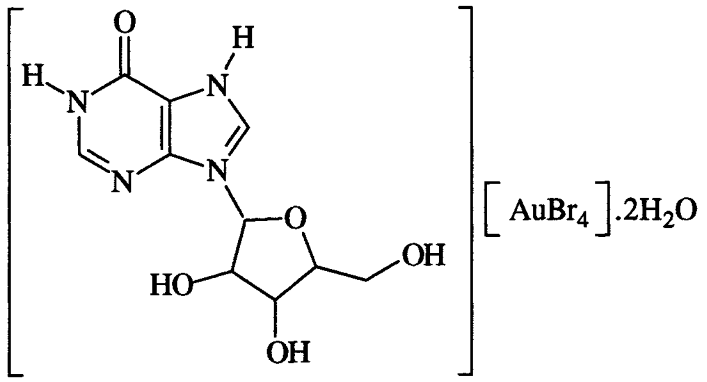

**Inosinium Tetrabromoaurate(III) Dihydrate**

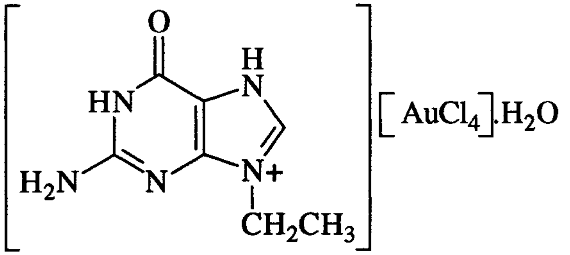

**9-Ethylguaninium Tetrachloroaurate(III) hydrate**

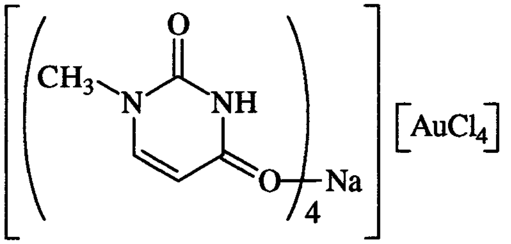

**Tetrakis(1-methyluracilium)sodiun Tetrachloroaurate(III)**

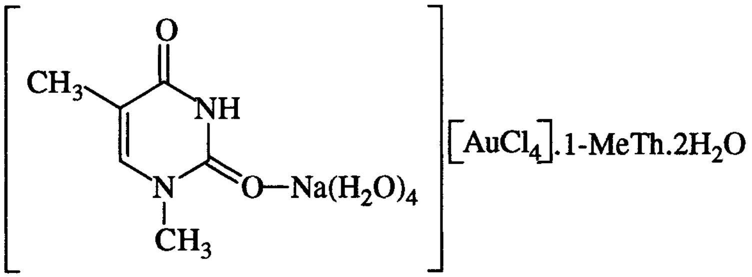

**$[Na(1\text{-}MeTh)(H_2O)_4][AuCl_4]$ $.1\text{-}MeTh.2H_2O$**

**Figure 7a**: Gold salts with nucleobases which have been studied by X-ray crystallography.

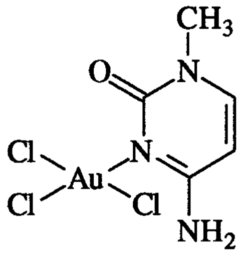

**Trichloro(1-methyl-cytosinato)gold(III)**

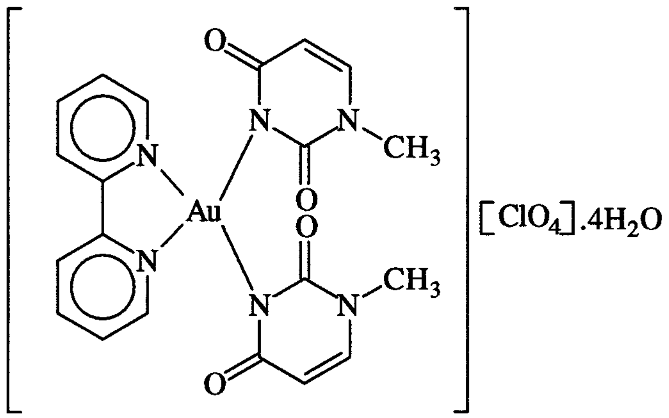

**[Au(2,2′-bipy)(1-MeU)$_2$]ClO$_4$.4H$_2$O**

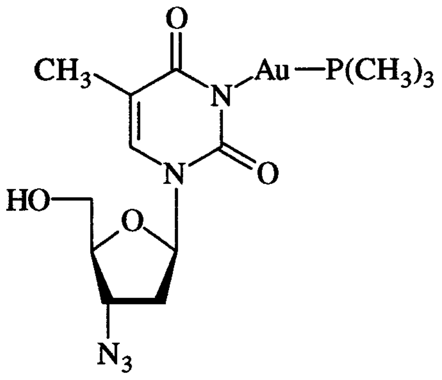

**[(CH$_3$)$_3$PAu(AZT)]**

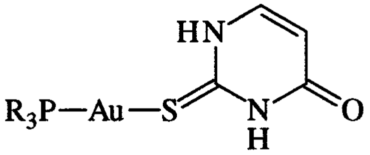

R = ethyl, phenyl

**Gold phosphine complexes with 2-thiouracil**

**Figure 7b**: Gold complexes of nucleobases which have been studied by X-ray crystallography.

### 4. POTENTIAL GOLD ANTITUMOUR DRUGS

### 4.1. Cytotoxic gold phosphines and other complexes

Auranofin, the antiarthritic drug, is potently cytotoxic to human cancer cells in culture[118] and also increases the survival time of mice with P388 leukemia.[119] However, it is active only against intraperitoneal (ip) P388 leukemia in mice, and then only when administered ip. It does not alter cell cycle distribution but DNA, RNA and protein syntheses are inhibited at concentrations that are lethal to cells.[90]

A large number of gold monodentate phosphine-thiolate complexes have since been tested for antitumour activity, with variation of both phosphine and thiolate ligands.[120] These are described in Table 2 with their anticancer activity data. Comparison of Au(I) thiolates with phosphine Au(I) thiolates suggests that it is the phosphine that is the toxic agent and that the metal plays a role in delivery of the complex to the target site. By comparison, the triethylarsine gold complex $Et_3AsAuCl$ shows potent cytotoxicity *in vitro* but is inactive *in vivo*.[120]

The activation energies for ligand exchange reactions on Au(I) through associative 3-coordinate intermediates are low. $^{31}P$ Nmr studies show that the Au(I) phosphine complexes undergo fast ligand exchange reactions with free phosphine at 213 K.[121] Four-coordinate tetrahedral Au(I) bisphosphine complexes undergo much slower ligand redistribution reactions. There is no evidence for 5-coordinate Au(I) complexes, hence all exchange reactions require ring-opening of the chelates. Four-coordinate complexes with 5- or 6-membered rings readily form, but bisphosphines with longer chains between phosphorus atoms form annular 3-coordinate Au(I) dimers or bridged digold species, with trigonal and linear coordination at gold, respectively.

Bridged linear digold(I) bisphosphine compounds (Figure 8) such as (thioglucose)Au(μ-dppe)Au(thioglucose) are also cytotoxic *in vitro*, and exhibit *in vivo* anticancer activity against a wider range of tumours compared to auranofin. A large number of analogues have been prepared and tested for their anticancer activity (Table 3).[122] The corresponding Au(III) complex, $[Cl_3Au(\mu\text{-}dppe)AuCl_3]$, also shows activity.[123] The structures of the bridged linear Au(I) complexes are assumed to be the same as [ClAu(μ-dppe)AuCl], which has linear 2-coordinate Au(I).[124]

### Table 2

Antitumour Activity Of Auranofin Analogues In Mice With Ip P388 Leukemia And In Vitro Cytotoxicity Against B16 Melanoma Cells.

| Structure | MTD[a] (μmol/kg/day) | %ILS[b] | $IC_{50}$[c] (μM) |
|---|---|---|---|
| $Et_3PAuSGlu(Ac)_4$ | 18 | 70 | 1.5 |
| $Et_3PAuGlu$ | 24 | 68 | 2 |
| $Et_3PAuSGlu(CONHCH_3)_4$ | 21 | 58 | 7 |
| $Et_3PAuSGlu(SO_2CH_3)_4$ | 19 | 40 | 3 |
| $Et_3PAuS$-α-$Glu(Ac)_4$ | 18 | 65 | 4 |
| $Et_3PAuSGal(Ac)_4$ | 18 | 88 | 4 |
| $Et_3PAuSGlu(Ac)_3Glu(Ac)_4$ | 19 | 88 | 6 |
| $Et_3PAuS$-(1-β-thio-2-isopropylidene-xylofuranose) | 15 | 27 | 6 |
| $Et_3PAuSCH_3$ | 110 | 36 | 60 |
| $Et_3PAuS(CH_2)_7CH_3$ | 17 | 32 | - |
| $Et_3PAuSCH(COOH)CH_2COOH$ | 22 | 46 | 1 |
| $Et_3PAuSCH_2CH_2N(CH_2CH_2)_2O.HCl$ | 24 | 32 | - |
| $Et_3PAuSCH_2CH_2OGlu$ | 14 | 55 | 2 |
| $Et_3PAuSCH_2CH_2SGlu$ | 21 | 45 | 7 |
| $Et_3PAuS$-glutathione.HCl | 12 | 32 | 2 |
| $Et_3PAuSCN$ | 21 | 36 | 1 |
| $Et_3PAuSC(NH)NH_2.HCl$ | 14 | 41 | 1 |
| $Et_3PAuSC(NH)NHNH_2.HCl$ | 11 | 36 | 1 |
| $Et_3PAuSPh$ | 19 | 36 | 6 |
| $Et_3PAuS$-(o-$H_2N$-$C_6H_4$) | 18 | 63 | 1 |

### Table 2 (cont.)

**Antitumour Activity Of Auranofin Analogues In Mice With Ip P388 Leukemia And In Vitro Cytotoxicity Against B16 Melanoma Cells.**

| Structure | MTD[a] (μmol/kg/day) | %ILS[b] | $IC_{50}$[c] (μM) |
|---|---|---|---|
| $Et_3PAuS$-(2-pyridyl) | 14 | 46 | 2 |
| $Et_3PAuS$-(4-pyridyl) | 9 | 36 | 1 |
|   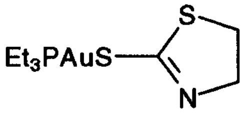 | 19 | 60 | 4 |
|   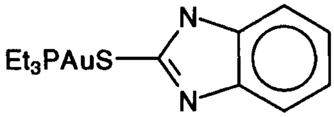 | 17 | 45 | 5 |
|   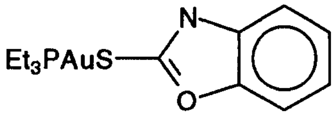 | 11 | 27 | 3 |
| $Et_3PAuS(EtOH)_2{}^+NO_3{}^-$ | 19 | 64 | 10 |
| $(CH_3)_3PAuSGlu(Ac)_4$ | 9 | 45 | 2 |
| $((CH_3)_2CH)_3PAuSGlu(Ac)_4$ | 14 | 46 | 4 |
| $((CH_3)_2N)_3PAuSGlu(Ac)_4$ | 8 | 60 | 2 |
| $Ph_3PAuSGlu(Ac)_4$ | 7 | 36 | 4 |
| $Et_2PhPAuSGlu(Ac)_4$ | 13 | 55 | 2 |
| $EtPh_2PAuSGlu(Ac)_4$ | 6 | 32 | 4 |
| $Et_2(EtO)PAuSGlu(Ac)_4$ | 14 | 70 | 1 |
| $Et_2((CH_3)_2CH)PAuSGlu(Ac)_4$ | 17 | 90 | 2 |
| $Et_2(HOBu)PAuSGlu(Ac)_4$ | 17 | 58 | 8 |
| $AuSGlu(Ac)_4$ | 110 | 14 | 150 |

### Table 2 (cont.)

Antitumour Activity Of Auranofin Analogues In Mice With Ip P388 Leukemia And In Vitro Cytotoxicity Against B16 Melanoma Cells.

| Structure | MTD[a] (μmol/kg/day) | %ILS[b] | $IC_{50}$[c] (μM) |
|---|---|---|---|
| AuSGlu | >300 | 15 | 166 |
| $AuSCH_2CH_2OH$ | 500 | 9 | 140 |
| $AuSCH(COOH)CH_2COOH$ | 350 | 24 | 60 |
| $AuSCH_2CH_2OGlu(Ac)_4$ | 106 | 0 | >100 |
| $Et_3PAuCl$ | 14 | 36 | 1 |
| $Et_3PAuCH_3$ | 36 | 55 | 1 |
| $Et_3PAuCN$ | 17 | 68 | 0.4 |
| $Et_3PAuNO_3$ | 13 | 41 | 2 |
| $(Et_3P)_2AuCl$ | 8 | 36 | 1 |
| $Et_3PAu(PPh_3)Cl$ | 13 | 52 | 6 |
| $(CH_3)_3PAuCl$ | 16 | 34 | 6 |
| $(allyl)_3PAuCl$ | 5 | 25 | 15 |
| $Et_2(HOBu)PAuCl$ | 15 | 27 | 8 |
| $((CH_3)_2N)_3PAuCl$ | 13 | 31 | 0.7 |
| $Ph_3PAuCl$ | 20 | 36 | 12 |
| $Et_2PhPAuCl$ | 8 | 41 | 1 |
| $((CH_3)_2CH)_2PhPAuCl$ | 9 | 36 | 3 |
| $(PhCH_2)_3PAuCl$ | 19 | 91 | 4 |
| $Ph(PhCH_2)_2PAuCl$ | 10 | 66 | 2 |

### Table 2 (cont.)

Antitumour Activity Of Auranofin Analogues In Mice With Ip P388 Leukemia And In Vitro Cytotoxicity Against B16 Melanoma Cells.

| Structure | MTD[a] (μmol/kg/day) | %ILS[b] | $IC_{50}$[c] (μM) |
|---|---|---|---|
| $Et_3AsAuCl$ | 40 | 18 | 8 |
| $Et_2SAuCl$ | 500 | 50 | >200 |
|  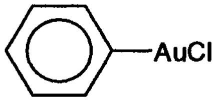 | 128 | 35 | 125 |
|  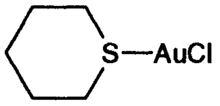 | 60 | 32 | 67 |
|  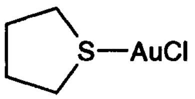 | 100 | 32 | 67 |
|  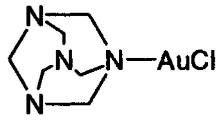 | 64 | 14 | 155 |
|  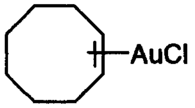 | 190 | 41 | 25 |

[a]Maximally tolerated dose for mice on an every day for 5 days regimen. [b]Maximum increase in life span produced in mice bearing P388 leukemia ip; figures are generally average values for 2 experiments. [c]Concentration which inhibits cloning efficiency of B16 melanoma cells by 50% . Abbreviations Et, ethyl; Ph, phenyl; Glu, glucose; Gal, galactose; Ac, acetate; Bu, butyl. (Data from Mirabelli et al., 1986).[120]

### Table 3

**Antitumour Activity Of Linear Bridged Diphosphine Complexes, [ClAu($R_2XYXR'_2$)AuCl] In Mice With Ip P388 Leukemia And In Vitro Cytotoxicity Against B16 Melanoma Cells.**

| Complex | | | MTD[a] (μmol/kg/day) | %ILS[b] | $IC_{50}$[c] (μM) |
|---|---|---|---|---|---|
| R, R' | Y | X | | | |
| Ph | $(CH_2)_2$ | P | 7 | 98±4 | 8 |
| Et | $(CH_2)_2$ | P | 60 | neg | 17 |
| *c*-$C_6H_{11}$ | $(CH_2)_2$ | P | 18 | 60, 80 | 14 |
| $PhCH_2$ | $(CH_2)_2$ | P | 4 | neg | 4 |
| 2-furyl | $(CH_2)_2$ | P | 5 | neg | 17 |
| 2-thienyl | $(CH_2)_2$ | P | 3 | neg | 6 |
| 2-pyridyl | $(CH_2)_2$ | P | 7 | 55, 60 | 4 |
| 4-pyridyl | $(CH_2)_2$ | P | 14 | neg | - |
| Ph, Et | $(CH_2)_2$ | P | 20 | neg | 2 |
| 4-F-$C_6H_4$ | $(CH_2)_2$ | P | 9 | 80, 75 | 8 |
| 2-F-$C_6H_4$ | $(CH_2)_2$ | P | 9 | 60, 55 | 6 |
| 4-tolyl | $(CH_2)_2$ | P | 4 | neg | 3 |
| 3-tolyl | $(CH_2)_2$ | P | 4 | neg | 7 |
| 4-$CF_3$-$C_6H_4$ | $(CH_2)_2$ | P | 7 | 37, 44 | - |
| 4-HO-$C_6H_4$ | $(CH_2)_2$ | P | 35 | neg | - |
| 4-$CH_3O$-$C_6H_4$ | $(CH_2)_2$ | P | 3 | neg | 7 |
| 2-$CH_3O$-$C_6H_4$ | $(CH_2)_2$ | P | 12 | 44, 79 | - |
| 4-$CH_3S$-$C_6H_4$ | $(CH_2)_2$ | P | 8 | 37, 65 | - |
| 2-$CH_3S$-$C_6H_4$ | $(CH_2)_2$ | P | 8 | 70±28 | - |

### Table 3 (cont.)

Antitumour Activity Of Linear Bridged Diphosphine Complexes, $[ClAu(R_2XYXR'_2)AuCl]$ In Mice With Ip P388 Leukemia And In Vitro Cytotoxicity Against B16 Melanoma Cells.

| Complex R, R' | Y | X | MTD[a] (µmol/kg/day) | %ILS[b] | $IC_{50}$[c] (µM) |
|---|---|---|---|---|---|
| $4\text{-}(CH_3)_2N\text{-}C_6H_4$ | $(CH_2)_2$ | P | 8 | neg | - |
| $C_6D_5$ | $(CH_2)_2$ | P | 7 | 40, 65 | 8 |
| Ph | $CH_2$ | P | 59 | 65, 50 | 6 |
| Ph | *cis*-CH=CH | P | 5 | 105, 77 | 7 |
| Ph | *trans*-CH=CH | P | 28 | 33, 36 | 10 |
| Ph | C≡C | P | 37 | neg | 23 |
| Ph | $CH_2CH(CH_3)$ | P | 7 | 43±6 | 4 |
| Ph | $CH(CH_3)CH(CH_3)$ | P | 7 | 44±15 | 17 |
| Ph | $(CH_2)_3$ | P | 7 | 62±13 | 2 |
| Ph | $(CH_2)_4$ | P | 7 | 40, 45 | 3 |
| Ph | $(CH_2)_5$ | P | 4 | 55, 55 | 2 |
| Ph | $(CH_2)_6$ | P | 4 | 40, 35 | 2 |
| Ph | $1,4\text{-}C_6H_4$ | P | 26 | neg | 13 |
| Ph | $(CH_2)_2$ | P, As | 9 | neg | 4 |
| Ph | $(CH_2)_2$ | As | 17 | neg | 7 |
| Ph | $(CH_2)_2$ | S | 90 | neg | 30 |

[a]Maximally tolerated dose for B6D2F1 mice on an every day for 5 days regimen. [b]Maximum increase in life span produced in mice bearing P388 leukemia ip; figures separated by commas represent different experiments; a drug is considered active if it produces >30% ILS. [c]Concentration which inhibits cloning efficiency of B16 melanoma cells by 50% on 2 h exposure. (Data from Mirabelli et al., 1987).[122]

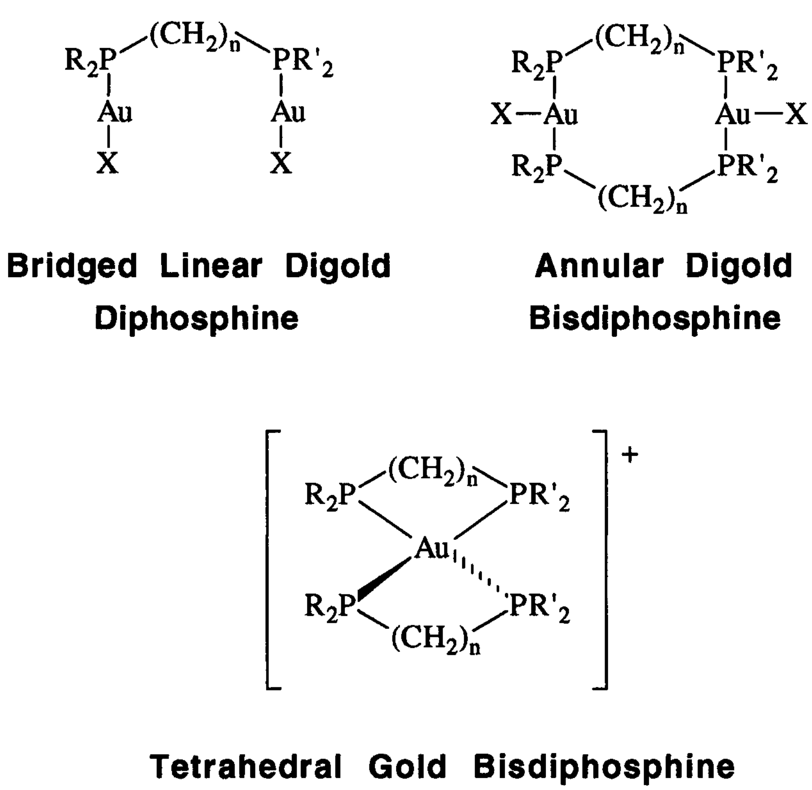

**Figure 8**: General structures of cytotoxic gold(I) diphosphine complexes.

Annular Au(I) complexes such as $[Au_2(Ph_2PCH_2CH_2PEt_2)_2Cl_2]$ have 3-coordinate trigonal gold centres which are probably stabilized by short Au···Au contacts (Figure 8).[125] The structure - activity relationships for this series indicate that the phosphine is important. Replacement of phosphorus with arsenic or sulfur results in inactivity, and the activity is optimized for 2-carbon bridges between the phosphorus atoms; also replacement of the phenyl group can alter the activity.[122, 123] These complexes are readily converted into tetrahedral complexes (Figure 8) in blood plasma, or on reaction with thiolates, sulfide, or further bisphosphine ligand.[126, 127] In these complexes Au(I) has a flattened tetrahedral geometry with average Au - P bond lengths of ca. 2.4 Å.[127, 128] The tetrahedral complexes exhibit surprisingly high kinetic and thermodynamic stability, with sharp $^{31}P$ nmr signals for free ligand and complex at room temperature. The mixed-ligand complex $[Au(dppe)(depe)]^+$, shows a $^{31}P$-$^{31}P$ coupling constant of 52 Hz, showing that the rate of ring opening is <52 $s^{-1}$.[128] Unlike auranofin, the tetrahedral complexes remain intact in human plasma and do not readily undergo ligand reactions with glutathione or albumin, and lipophilic tetrahedral complexes such as $[Au(dppe)_2]^+$ readily partition into lipoproteins or bind within cell membranes.[129]

$[Au(dppe)_2]^+$ is highly toxic to tumour cells *in vitro*, although cells can exhibit some resistance at low doses. The complex was found to be active to a P388 subline that was resistant to cisplatin. Moreover, administration of a combination of cisplatin and $[Au(dppe)_2]Cl$ was more effective against P388 leukemia than either agent alone.[130] The primary cytotoxic lesion appears to involve DNA-protein crosslinks, although DNA strand-breaks also occur at higher doses. The free diphosphine ligand, dppe, is also active against P388 leukaemina but is much less potent, suggesting that part of the action of Au(I) is to protect the ligand from oxidation reactions before it reaches the target site. In the series of diphosphine ligands $Ph_2P(CH_2)_nPPh_2$, activity is highest for n = 2 and 3 or where the bridge between the phosphorus atoms is *cis*-CH=CH, i.e. for those ligands which can form strong chelate rings. In the series of tetrahedral Au(I) complexes $[Au(R_2PCH_2CH_2PR'_2)_2]^+$ antitumour activity is gradually lost on replacing R or R′ = Ph with Et.[131] Table 4 lists some of the analogues which have been tested for cytotoxicity, although these have lower activities than the dppe complex. It is possible that chelation of the free ligand to Cu(I) inside cells is essential for activity. Cu(I) and Ag(I) dppe complexes are known to be active.[123, 130, 131]

Arsenic analogues, such as the bridged digold and tetrahedral gold complexes of the ligand dadpe ($Ph_2PCH_2CH_2AsPh_2$) have been tested against 3 cell lines: L1210 (antimetabolite-sensitive leukemia cells), WS (alkylating-agent-sensitive Walker tumour cells) and V.79 (Chinese hamster lung cells). They are both cytotoxic to all 3 cell lines, although the tetrahedral complex is more toxic to V.79 cells, while the corresponding dppe complex is 8 times more toxic to L1210 cells. The lower potency compared to diphosphines is probably a reflection of the increased kinetic lability and lower thermodynamic stability of Au-As bonds compared to Au-P bonds, and hence the ligand is more easily displaced and detoxified by oxidation.[132]

In contrast to $R_3PAuX$ complexes in the auranofin series, the antitumour activity of the bridged digold complexes increases when X is a good leaving group (with $R_3PAuX$ complexes good leaving groups result in binding to serum proteins such as albumin). This is probably related to the conversion of the linear bridged complexes to the kinetically stable tetrahedral complexes. The major drawback to the clinical use of tetrahedral complexes such as $[Au(dppe)_2]^+$ is their ability to uncouple mitochondrial oxidative phosphorylation by increasing the permeability of the mitochondrial membrane to cations, with collapse of the mitochondrial membrane potential.[133] In preclinical trials, $[Au(dppe)_2]^+$ exhibited cardiac, hepatic and vascular toxicity.[134, 135]

### Table 4

Antitumour Activity Of Tetrahedral Gold Bisdiphosphine Complexes, $[Au(R_2PYPR'_2)_2]X$, In Mice With Ip P388 Leukemia And In Vitro Cytotoxicity Against B16 Melanoma Cells.

| Complex | | | MTD[a] (μmol/kg/day) | %ILS[b] | $IC_{50}$[c] (μM) |
|---|---|---|---|---|---|
| R, R' | Y | X | | | |
| Ph | $(CH_2)_2$ | Cl | 3 | 83±25 | 4.5 |
| Ph | $(CH_2)_2$ | Br | 2 | 70, 83 | - |
| Ph | $(CH_2)_2$ | I | 2 | 60, 150 | - |
| Ph | $(CH_2)_2$ | $NO_3$ | 3 | 90±17 | 4 |
| Ph | $(CH_2)_2$ | $CH_3SO_3$ | 2 | 81±10 | - |
| Ph | $(CH_2)_2$ | $HO(CH_2)_2SO_3$ | 2 | 55, 78 | - |
| Ph | $(CH_2)_3$ | Cl | 3 | 89+28 | 0.6 |
| Ph | *cis*-CH=CH | Cl | 2 | 92±26 | 2 |
| 3-fluorophenyl | $(CH_2)_2$ | Cl | 10 | 45, 55 | - |
| 4-fluorophenyl | $(CH_2)_2$ | Cl | 3 | 55, 50 | - |
| Ph, Et | $(CH_2)_2$ | Cl | 4 | 54±16 | 5 |
| 2-pyridyl | $(CH_2)_2$ | Cl | 8 | 75±5 | - |
| 4-pyridyl | $(CH_2)_2$ | Cl | 6 | inactive | - |
| Et | $(CH_2)_2$ | $PF_6$ | 5 | 40, 30 | 17 |
|  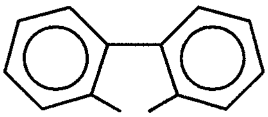 | $(CH_2)_2$ | $NO_3$ | 4 | 61, 33 | - |

[a]Maximally tolerated dose for B6D2F1 mice on an every day for 5 days regimen. [b]Maximum increase in life span produced in mice bearing P388 leukemia ip; figures separated by commas represent different experiments; a drug is considered active if it produces >30% ILS. [c]Concentration inhibiting cloning efficiency of B16 melanoma cells by 50% on 2 h exposure. (Data from Berners-Price et al., 1990).[131]

There are a few reports of non-phosphine Au(III) complexes with anticancer activity. These include $[Au(CN)_2]^-$, $[Au(III)Me_2Cl_2]AsPh_4$, $[Me_2Au(III)(SCN)_2Au(III)Me_2]$[136] and $[Au(dedetc)Br_2]$, (dedtc = diethyldithiocarbamate).[137] In contrast to Pt(II) compounds, these active compounds have tightly bound (and potentially toxic) ligands with high *trans* influences. Colloidal radioactive $^{198}Au$ is reported to protect rats against hepatic tumour growth.[138] However, despite these difficulties, gold(I) phosphine complexes show promise as antitumour agents, particularly as complements to other metal drugs already in clinical use, and there remains much to be explored in their development.

### References

1. Sadler, P.J., *Gold Bull.*, **1976**, 110, 10.
2. Sutton, B.M., *Gold Bull.*, **1986**, 19, 15.
3. D'Arcy Hart, P., *Brit. Med. J.*, **1946**, Nov 30, 805 and Dec 7, 849.
4. Forestier, J., *Bull. Mém. Soc. Méd. Hôp. (Paris)*, **1929**, 53, 3231.
5. Shaw III, C.F., *Inorganic Perspectives in Medicine and Biology*, **1979**, 2, 287.
6. Brown, D.H. and Smith, W.E., *Chem. Rev.*, **1980**, 9, 217.
7. Berners-Price, S.J. and Sadler, P.J., *Gold Drugs* in Frontiers In Bioinorganic Chemistry, Xavier, A.V. (Ed.), VCH Publ. Weinheim, 1986, pp 376-388.
8. Champion, G.D., Graham, G.G., Ziegler, J.B., *Balliéré's Clin. Rheumatol.*, **1990**, 4, 491.
9. Dash, K.C. and Schmidbauer, H., *Metal Ions In Biol. Syst.*, **1983**, 14, 180.
10. Smith, W.E. and Reglinski, J., *Perspectives In Bioinorganic Chemistry*, **1991**, 1,183.
11. Parish, R.V., *Interdisciplinary Science Reviews*, **1992**, 17, 221.
12. Ni Dhubhghaill, O.M. and Sadler, P.J., *Gold Complexes In Cancer Chemotherapy* in Metal Complexes In Chemotherapy, Keppler, B.K. (Ed.), VCH Publ., Weinheim, 1993, pp 225-250.
13. Puddephatt, R.J., The Chemistry Of Gold, Elsevier, Amsterdam, 1976.
14. Beesley, J.E., Colloidal Gold: A New Perspective For Cytochemical Marking, Oxford University Press, Oxford, 1989.
15. Yeung, S.A., Hobson, R., Biggs, S. and Grieser, F., *J. Chem. Soc. Chem. Commun.* **1993**, 378.
16. Duff, D.G., Baiker, A. and Edwards, P.P., *J. Chem. Soc. Chem. Commun.*, **1993**, 96.
17. Al-Sa'ady, A.K.H., McAuliffe, C.A., Moss, K., Parish, R.V. and Fields, R., *J. Chem. Soc. Dalton Trans.*, **1984**, 491 (and refs. therein).

18. Elder, R.C. and Eidsness, M.K., *Chem. Rev.*, **1987**, 87, 1027.

19. Elder, R., Zhao, Z., Zhang, Y., Dorsey, J.G., Hess, E.V. and Tepperman, K., *J. Rheum.*, **1993**, 20, 268.

20. Beverley, B. and Couri, D., *Federation Proceedings*, **1987**, 46, 854.

21. Hawkins, C.J., Moensted, O. and Bjerrum, J., *Acta Chem. Scand.*, **1970**, 24, 1059.

22. Sadler, P.J., *J. Rheum.*, **1982**, 9 (suppl. 8), 71.

23. Kissner, R. Latal, P and Geier, G., *J. Chem Soc. Chem. Commun.*, **1993**, 136.

24. Pathaneni, S.S. and Desiraju, G.R., *J. Chem. Soc. Dalton Trans.*, **1993**, 319.

25. Landé, K. (1927). *Münch. Med. Wchschr.*, **1927**, 7, 1132.

26. Ruben, H., Zalkin, A., Faltens, M.O. and Templeton, D.H., *Inorg. Chem.*, **1974**, 13, 1836.

27. Mazid, M.A., Razi, M.T., Sadler, P.J., Greaves, G.N., Hurman, S.J., Koch, M.H.J. and Phillips, J.C., *J. Chem. Soc. Chem. Commun.*, **1980**, 1261.

28. Elder, R.C., Eidsness, M.K., Heeg, M.J., Tepperman, K.G., Shaw III, C.F. and Schaeffer, N., *ACS. Symposium Series*, **1983**, 209, 385.

29. Isab, A.A. and Sadler, P.J., *J. Chem. Soc. Dalton Trans.*, **1981**, 1657.

30. Elder, R. Ludwig, K., Cooper, J.N. and Eidsness, M.K., *J. Am. Chem. Soc.*, **1985**, 107, 5024.

31. Grootveld, M.C., Razi, M.T. and Sadler, P.J., *Clinical Rheum.*, **1984**, 3 (suppl. 1), 5.

32. Grootveld, M.C. and Sadler, P.J., *J. Inorg. Biochem.*, **1983**, 19, 51.

33. Vernon-Roberts, B., Dore, J.L., Jessop, J.D. and Henderson, W.J., *Ann. Rheum. Dis.*, **1976**, 35, 477.

34. Walz, D.T., DiMartino, M.J., Sutton, B.M. and Misher, A.,*J. Pharm. Exp. Ther.*, **1972**, 181, 292.

35. Hill, D.T., U.S. Patent: 4,057,630 - *Chem. Abs.*, **1978**, 88, 94838w.

36. Hill, D.T. and Sutton, B.M., *Cryst. Struct. Comm.*, **1980**, 9, 679.

37. Razi, M.T., Sadler, P.J., Hill, D.T. and Sutton, B.M., *J. Chem. Soc. Dalton Trans.*, **1983**, 1331.

38. Razi, M.T., Otiko, G. and Sadler, P.J., *ACS Symposium Series*, **1983**, 209, 371.

39. Tiekink, E.R.T., *Acta Cryst.*, **1989**, C45, 1233.

40. Cottrill, S.M., Sharma, H.L., Dyson, D.B., Parish, R.V.and McAuliffe, C.A., *J. Chem. Soc. Perkin Trans. II*, **1989**, 53.

41. Finkelstein, A.E., Burrone, O.R., Walz, D.T. and Misher, A., J. Rheum., **1977**, 4, 245.

42. Froscio, M., Murray, A.W. and Hurst, N.P., *Biochem. Pharm.*, **1989**, 38, 2087.

43. Mahoney, C.W., Hensey, C.E. and Azzi, A., *Biochem. Pharm.*, **1989**, 38, 3383.

44. Corey, E.J., Mehrotra, M.M. and Khan, A.U., *Science*, **1987**, 236, 68.

45. Grootveld, M., Blake, D.R., Sahinoglu, T., Claxson, A.W.D., Mapp, P. Stevens, C., Allen, R.E. and Furst, A., *Free Rad. Res. Comms.*, **1990**, 10, 199.
46. Sutton, B.M. and Weinstock, J., U.S. Patent: 3,903,274 - *Chem. Abs.*, **1976**, 84, 17559.
47. Vinson, J., Mathewson, E., Kelly, J., Yang, W., Bennett, J., Hanchak, N., Farrell, N. and Lupia, J., *Int. Cong. Pac. Basin Socs.*, **1989**, ACS Abs. BIOS, 130.
48. Isab, A.A. and Sadler, P.J., *J. Chem. Soc. Dalton Trans.*, **1982**, 135.
49. Thompson, R.H.S. and Whittaker, V.P., *Biochem. J.*, **1947**, 41,342.
50. Hill, D.T., *Med. Clin. N. Amer.*, **1968**, 52, 733.
51. Shaw III, C.F., Schaeffer, N.A. Elder, R.C., Eidsness, M.K. Trooster, J.M. and Calis, G.H.M., *J. Am. Chem. Soc.*, **1984**, 106, 3511.
52. Smith, W.E., Reglinski, J., Hoey, S., Brown, D.H. and Sturrock, R.D., *Inorg. Chem.*, **1990**, 29, 5190.
53. Isab, A.A., Ghazi, I.H., Wazeer, M.I.M. and Perzanowski, H.P., *J. Inorg. Biochem.*, **1993**, 50, 299.
54. Tepperman, K., Finer, R., Donovan, S., Elder, R.C., Doi, J., Ratliff, D. and Ng, K., *Science*, **1984**, 225, 430.
55. Malik, N.A. and Sadler, P.J., *Biochem. Soc. Trans.*, **1979**, 7, 731.
56. Shaw III, C.F., *Comm. Inorg. Chem.*, **1989**, 8, 233.
57. Shaw III, C.F., Coffer, M.T., Klingbeil, J. and Mirabelli, C.K., *J. Am. Chem. Soc.*, **1988**, 110, 729.
58. Kinsch, E.M. and Stephan, D.W., *Inorg. Chim. Acta*, **1984**, 91, 263.
59. Otiko, G. and Sadler, P.J., *FEBS Lett.*, **1980**, 116, 227.
60. Intoccia, A.P., Flanagan, T.L., Walz, D.T., Gutzait, L., Swagzdis, J.E., Flagiello Jr., J., Hwang, Y.-H. B., Dewey, R.H. and Noguchi, H., *J. Rheum.*, **1982**, 9 (suppl. 8), 90.
61. Malik, N.A., Otiko, G. and Sadler, P.J., *J. Inorg. Biochem.*, **1980**, 12, 317.
62. Graham, G.G., Haavisto, T,M. and McNaught, P.J., *J. Rheum.*, **1982**, 9, 527.
63. Graham, G.G., Bales, J.R., Grootveld, M.C. and Sadler, P.J., *J. Inorg. Biochem.*, **1985**, 25, 163.
64. Finkelstein, A.E., Walz, D.T., Batista, V., Mizraji, M., Toisman, F. and Misher, A., *Ann. Rheum. Dis.*, **1976**, 35, 251.
65. Coffer, M.T., Shaw III, C.F., Eidsness, M.K., Watkins II, J.W. and Elder, R.C., *Inorg. Chem.*, **1986**, 25, 333.
66. Ecker, D.J., Hempel, J.C., Sutton, B.M., Kirsch, R. and Crooke, S.T., *Inorg. Chem.*, **1986**, 25, 3139.
67. Ni Dhubhghaill, O.M. , Sadler, P.J. and Tucker, A., *J. Am. Chem. Soc.*, **1992**, 114, 1118.

68. Xiao, J. and Shaw III, C.F. (1992). *Inorg. Chem.*, **1992**, 31, 3706.
69. Isab, A.A., Hormann, A.L., Coffer, M.T. and Shaw III, C.F., *J. Am. Chem. Soc.*, **1988**, 110, 3278.
70. Isab, A.A., Shaw III, C.F. and Locke, J., *Inorg. Chem.*, **1988**, 27, 3406.
71. Isab, A.A., Hormann, A.L., Hill, D.T., Griswold, D.E., DiMartino, M.J. and Shaw III, C.F., *Inorg. Chem.*, **1989**, 28, 1321.
72. Coffer, M.T., Shaw III, C.F., Hormann, A.L., Mirabelli, C.K. and Crooke, S.T., *J. Inorg. Biochem.*, **1987**, 30, 177.
73. Laib, J.E., Shaw III, C.F., Petering, D.H., Eidsness, M.K., Elder, R.C. and Garvey, J.S., *Biochemistry*, **1985**, 24, 1977.
74. Shaw III, C.F. and Laib, J.E., *Inorg. Chim. Acta*, **1986**, 123, 197.
75. Glennas, A. and Rugstadt, H.E., *J. Rheum.*, **1985**, 14, 25.
76. Butt, T.R., Sternberg, E.J., Mirabelli, C.K. and Crooke, S.T., *Mol. Pharm.*, **1986**, 29, 204.
77. Mogilnicka, E.W. and Webb, M., *Biochem. Pharm.*, **1983**, 32, 1341.
78. Witkiewicz, P.L. and Shaw III, C.F., *J. Chem. Soc. Chem. Commun.*, **1981**, 1111.
79. Wienken, M., Lippert, B., Zangrando, E. and Randaccio, L., *Inorg. Chem.*, **1992**, 31, 1983.
80. Anderson, J.E. and Sawtelle, S.M., *Inorg. Chim. Acta*, **1992**, 194, 171.
81. Ambach, E., Singh, M.M., Nagel, U. and Beck, W., *Z. Scand.*, **1984**, 24, 3567.
82. Jones, P.G. and Schelbach, R.,*Inorg. Chim. Acta*, **1991**, 182, 239.
83. Pinkerton, M., Steinrauf, L.K. and Dawkins, P., *Biochem. Biophys. Res. Comm.*, **1969**, 35, 512.
84. Söderberg, B.O., Zeppezauer, E., Boive, T., Nordström, B. and Brädén, C.I., *Acta. Chem. Scand.*, **1970**, 24, 3567.
85. Schuhmann, D., Kubicka-Muranyi, M., Mirtschewa, J., Günther, J. and Gleichmann, E.,*J. Immunol.*, **1990**, 145, 2132.
86. Romagnoli, P., Spinas, G.A. and Sinigaglia, F., *J. Clin. Invest.*, **1992**, 89, 254.
87. Ranford, J.D., Rhodes, M.D. and Sadler, P.J., *Metallodrugs* in Metallothioneins: synthesis, structure and properties of metallothioneins, phytochelatins and metal thiolate complexes, Stillman, M.J., Shaw III, C.F. and Suzuki, K.T. (Eds.), VCH Publ., New York, 1991, pp 414-416.
88. Handel, M.L., deFazio, A., Watts, C.K.W., Day, R.O. and Sutherland, R.L., *Mol. Pharm.*, **1991**, 40, 613.
89. Allaudeen, H.S., Snyder, R.M., Whitman, M.H. and Crooke, S.T., *Biochem. Pharm.*, **1985**, 34, 3243.

90. Mirabelli, C.K., Johnson, R.K., Sung, C.M., Faucette, L.F., Muirhead, K. and Crooke, S.T., *Cancer Res.*, **1985**, 45, 32.

91. Nechay, B.R., *Arth. and Rheum.*, **1980**, 23, 464.

92. Peters-Golden, M. and Shelly, C., *Biochem. Pharm.*, **1989**, 38, 1589.

93. Caltabiano, M.M., Poste, G. and Greig, R.G., *Biochem. Pharm.*, **1988**, 37, 4089.

94. Mirabelli, C.K., Sung, C.M., Zimmerman, J.P., Hill, D.T., Mong, S. and Crooke, S.T., *Biochem. Pharm.*, **1986**, 35, 1427.

95. Blank, C.E. and Dabrowiak, J.C., *J. Inorg. Biochem.*, **1984**, 21, 21.

96. Ward, B. and Dabrowiak, J.C., *J. Am. Chem. Soc.*, **1987**, 109, 3810.

97. Mizuno, Y. and Komiya, S., *Inorg. Chim. Acta*, **1986**, 125, L13.

98. Mizuno, Y. and Komiya, S., *Chem. Lett.*, **1986**, 1477.

99. Komiya, S., Mizuno, Y. and Shibuya, T., *Chem. Lett.*, **1988**, 367.

100. Ettorre, R., *J. Chem. Soc. Dalton Trans.*, **1983**, 2329.

101. Ettorre, R. and Martelli, M., *Inorg. Chim. Acta*, **1985**, 108, 73.

102. Ettorre, R. and González, A.S., *Inorg. Chim. Acta*, **1990**, 168, 221.

103. Kivekäs, R., Colacio, E., Ruiz, J., López-González, J.D. and Léon, P., *Inorg. Chim. Acta*, **1989**, 159, 103.

104. Dragulescu, C., Heller, J., Maurer, A., Policec, S., Topcui, V., Csalci, M., Kirschner, S., Kravitz, S. and Moraski, R. (1974). *Int. Coord. Chem. Conf. XVI*, **1974**, 1.9.

105. Salas, J.M., Quirós, M., Sánchez, M.P. and Beauchamp, A.L., *Acta Cryst.*, **1987**, C45, 1874.

106. Preut, H., Fischer, B. and Lippert, B., *Acta Cryst.* **1990**, C46, 1115.

107. Fischer, B., Preut, H., Lippert, B., Schöllhorn, H. and Thewalt, U., *Polyhedron*, **1990**, 9, 2199.

108. Holowczak, M.S., Stancl, M.D. and Wong, G.B., *J. Am. Chem. Soc.*, **1985**, 107, 5789.

109. Bressan, M., Ettorre, R. and Rigo, P., *J. Magn. Res.*, **1977**, 26, 43.

110. Micklitz, W., Lippert, B., Müller, G., Mikulcik, P. and Riede, J., *Inorg. Chim. Acta*, **1989**, 165, 57.

111. Pill, T., Polborn, K., Kleinschmidt, A., Erfle, V., Breu, W., Wagner, H. and Beck, W., *Chem. Ber.*, **1991**, 124, 1541.

112. Hoskins, B.F., Zhenrong, L. and Tiekink, E.R.T., *Inorg. Chim. Acta*, **1989**, 158, 7.

113. Harker, C.S.W., Tiekink, E.R.T. and Whitehouse, M.W., *Inorg. Chim. Acta*, **1991**, 181, 23.

114. Mirabelli, C.K., Zimmerman, J.P., Bartus, H.R., Sung, C.M., and Crooke, S.T., *Biochem. Pharm.*, **1986**, 35, 1435.

115. Gibson, D.W., Beer, M. and Barnett, R.J., *Biochemistry*, **1971**, 10, 3669.

116. Hadjiliadis, N., Pneumatikakis, G. and Basosi, R., *J. Inorg. Biochem.*, **1981**, 14, 115.
117. Calis, G.H.M. and Hadjiliadis, N., *Inorg. Chim. Acta*, **1984**, 91, 203.
118. Simon, T.M., Kunishima, D.H., Vibert, G.J. and Lorber, A., *Cancer*, **1979**, 44, 1965.
119. Simon, T.M., Kunishima, D.H., Vibert, G.J. and Lorber, A., *Cancer Res.*, **1981**, 41, 94.
120. Mirabelli, C.K., Johnson, R.K., Hill, D.T., Faucette, L.F., Girard, G.R., Kuo, G.Y., Sung, C.M. and Crooke, S.T., *J. Med. Chem.*, **1986**, 29, 218.
121. Mays, M.J. and Vergnano, P.A., *J. Chem. Soc. Chem. Commun.*, **1979**, 1112.
122. Mirabelli, C.K., Hill, D.T., Faucette, L.F., McCabe. F.L., Girard, G.R., Bryan, D.B., Sutton, B.M., O'Leary-Bartus, J., Crooke, S.T. and Johnson, R.K., *J. Med. Chem.*, **1987**, 30, 2181.
123. Snyder, R.M., Mirabelli, C.K., Johnson, R.K., Sung, C.M., Faucette, L.F., McCabe, F.L., Zimmerman, J.P., Whitman, M., Hempel, J.C. and Crooke, S.T., *Cancer Res.*, **1986**, 46, 5054.
124. Bates, P.A. and Waters. J.M., *Inorg. Chim. Acta*, **1985**, 98, 125.
125. Berners-Price, S.J. and Sadler, P.J., *Inorg. Chem.*, **1986**, 25, 3822.
126. Berners-Price, S.J., Jarrett, P.S. and Sadler, P.J., *Inorg. Chem.*, **1987**, 26, 3074.
127. Bates, P.A. and Waters. J.M., *Inorg. Chim. Acta*, **1984**, 81, 151.
128. Berners-Price, S.J., Mazid, M.A. and Sadler, P.J., *J. Chem. Soc. Dalton Trans.*, **1984**, 969.
129. Berners-Price, S.J. and Sadler, P.J., *J. Inorg, Biochem.*, **1987**, 31, 267.
130. Berners-Price, S.J., Mirabelli, C.K., Johnson, R.K., Mattern, M.R., McCabe, F.L., Faucette, L.F., Sung, C.M., Mong, S-M., Sadler, P.J. and Crooke, S.T., *Cancer Res.*, **1986**, 46, 5486.
131. Berners-Price, S.J., Girard, G.R., Hill, D.T., Sutton, B.M., Jarrett, P.S., Faucette, L.F., Johnson, R.K., Mirabelli, C.K. and Sadler, P.J., *J. Med. Chem.*, **1990**, 33, 1386.
132. Ni Dhubhghaill, O.M. , Sadler, P.J. and Kuroda, R., *J. Chem Soc. Dalton Trans.*, **1990**, 2913.
133. Hoke, G.D., Rush, G.F., Bossard, G.E., McArdle, J.V., Jensen, B.D. and Mirabelli, C.K., *J. Biol. Chem.*, **1988**, 263, 11203.
134. Rush, G.F., Alberts, D.W., Meunier, P., Leffler, K. and Smith, P.F., *Toxicologist*, **1987**, 7, 59.
135. Hoke, G.D., Macia, R.A., Meunier, P.C., Bugelski, P.J., Mirabelli, C.K., Rush, G.F. and Matthews, W.D., *Toxicol. and Appl. Pharm.*, **1989**, 100, 293.
136. Sadler, P.J., Nasr, M., Narayanan, V.L., in Platinum Coordination Complexes in Cancer Chemotherapy, Hacker, M.P., Douple, E.B. and Krakoff, I.H. (Eds.), Martinus Nijhoff Pub., Boston, 1984, 290-304.
137. Ni Dhubhghaill, O.M. and Sadler, P.J., unpublished results.

138. Alfonso, A.E., Hassan, A., Gardner, B., Stein, S., Patti, J., Solomon, N.A., McCarthy, J. and Steigman, J., *Cancer Res.*, **1978**, 38, 2740.

139. Sadler, P.J., *Struct. and Bond.*, **1976**, 29, 171.

**Received: August 12, 1993**

